# Lower Extremity Ulcers in Systemic Sclerosis: Features and Response to Therapy

**DOI:** 10.1155/2010/747946

**Published:** 2010-08-18

**Authors:** Victoria K. Shanmugam, Patricia Price, Christopher E. Attinger, Virginia D. Steen

**Affiliations:** ^1^Division of Rheumatology, Immunology and Allergy, Georgetown University Hospital, 3800 Reservoir Road, N.W., Hington, DC 20007, USA; ^2^Centre for Biomedical Sciences, Cardiff School of Health Sciences, University of Wales Institute-Cardiff, Western Avenue, Cardiff CF5 2YB, UK; ^3^Center for Wound Healing, Georgetown University Hospital, Washington, DC 20007, USA

## Abstract

Nondigital lower extremity ulcers are a difficult to treat complication of scleroderma, and a significant cause of morbidity. The purpose of this study was to evaluate the prevalence of nondigital lower extremity ulcers in scleroderma and describe the associations with autoantibodies and genetic prothrombotic states. A cohort of 249 consecutive scleroderma patients seen in the Georgetown University Hosptial Division of Rheumatology was evaluated, 10 of whom had active ulcers, giving a prevalence of 4.0%. Patients with diffuse scleroderma had shorter disease duration at the time of ulcer development (mean 4.05 years ± 0.05) compared to those with limited disease (mean 22.83 years ± 5.612, *P* value .0078). Ulcers were bilateral in 70%. In the 10 patients with ulcers, antiphospholipid antibodies were positive in 50%, and genetic prothrombotic screen was positive in 70% which is higher than expected based on prevalence reports from the general scleroderma population. Of patients with biopsy specimens available (*n* = 5), fibrin occlusive vasculopathy was seen in 100%, and all of these patients had either positive antiphospholipid antibody screen, or positive genetic prothrombotic profile. We recommend screening scleroderma patients with lower extremity ulcers for the presence of anti-phospholipid antibodies and genetic prothrombotic states.

## 1. Introduction

Non-digital lower extremity ulcers are a difficult to treat complication of scleroderma seen both in limited and diffuse scleroderma and also in scleroderma sine scleroderma. They contribute to the pain and disability of advanced disease. The etiology of these ulcers is unknown, but they may reflect chronic vasculopathy. 

The prevalence of nonhealing lower extremity ulcers in scleroderma has not specifically been studied. Older data from the Pittsburgh Scleroderma Databank identified seven out of 1030 patients requiring amputation for refractory leg ulcers, giving an incidence of wounds requiring amputation of 0.67% [1]. However, this is likely to be an underestimate of total prevalence of leg ulcers, since most scleroderma patients with leg ulcers do not require amputation. More recently, Alivernini et al. evaluated 130 scleroderma patients over a 20-month period. They identified 26.15% with digital ulcers and 3.8% with “other” ulcers [2], and the latter may be a more accurate estimate of the true prevalence of non-digital lower extremity ulcers. 

The impact of leg ulceration on health care costs and quality of life has not been studied in the scleroderma population. Extrapolating from other chronic diseases such as diabetes, it is known that leg ulcers result in significant morbidity and mortality, leading to recurrent hospitalizations, repeated surgeries, and significant costs to the health care system. A retrospective study of patients with diabetes and leg ulcers found that, in the first two years after diagnosis, the costs attributable to the ulcer were $27,987 [3]. 

Scleroderma is associated with delayed wound healing [2], and as with other chronic leg ulcers, the etiology of delayed healing is likely to be multifactorial. Some have postulated a role for larger vessel venous and arterial disease [4], but many scleroderma ulcers remain refractory even after restoration of good blood flow and venous drainage. 

Biopsy data from scleroderma wounds demonstrates fibrin plugging of the small vessels and persisting macrophage and fibroblast activation, suggesting that these wounds may be arrested in a chronic inflammatory phase. A similar fibrin occlusive vasculopathy is seen in biopsies of leg ulcers due to livedoid vasculopathy. Livedoid vasculopathy is associated with impaired fibrinolysis from a variety of genetic and acquired causes [5–7] and heparin an anticoagulant with profibrinolytic actions has been effective in some cases [8–10]. We postulate that dysregulation of the complement and coagulation cascades with inadequate fibrinolysis and angiogenesis may contribute to delayed healing in scleroderma- associated lower extremity ulcers. 

In autoimmune diseases, antiphospholipid antibodies are recognized as activators of both coagulation and complement cascades [11, 12]. Preliminary data in our connective tissue disease population has suggested an association between autoimmune ulcers and both antiphospholipid antibodies and genetic prothrombotic states [13]. 

The primary aim of the current study was to evaluate the prevalence of lower extremity ulcers in our scleroderma population. The secondary aim of this study was to evaluate the presence of antiphospholipid antibodies and genetic prothrombotic states in patients with scleroderma-associated leg ulcers. The outcomes of empiric therapy in the small number of patients evaluated in this study are reported. 

## 2. Methods

This study was approved by the Biomedical Institutional Review Board at Georgetown University Medical Center as part of the Connective Tissue Disease Leg Ulcer Etiology (CLUE) study. 

### 2.1. Patient Selection for Prevalence Evaluation

All scleroderma patients followed in the Georgetown University Hospital Division of Rheumatology and Wound Healing Center between August 2007 and August 2009 were evaluated for the presence of non-digital lower extremity ulcers. Active leg ulceration was defined as presence of non-digital lower extremity wounds that have been refractory to standard wound care for more than 3 months.

### 2.2. Laboratory Studies

Patients with active ulcers underwent autoimmune testing including antinuclear antibody by immunofluorescence (ANA), anti-Scl70 antibody, anticentromere antibody, antidouble stranded DNA antibody (dsDNA), anti-Sm antibody (Sm), anti-U1-RNP antibody (RNP), anti-Ro antibody (SSA), anti-La antibody (SSB), rheumatoid factor, and anticyclic citrullinated peptide. Prothrombotic evaluation was also completed in all patients including prothrombin gene mutation, plasminogen activator inhibitor-I mutation (PAI-I), methyltetrahydrofolate reductase mutation (MTHFR C677T), Factor V Leiden (FVL), protein S functional activity, protein C functional activity, antithrombin III functional activity, anticardiolipin IgG, IgA, and IgM titers, anti-*β*2-glycoprotein I IgG, IgA and IgM titers, and lupus anticoagulant. 

### 2.3. Venous and Arterial Testing

All patients had evaluation of ankle-brachial pressure index and venous Doppler ultrasound studies to identify any concomitant venous or arterial disease. If present, patients were referred to vascular surgery for therapy.

### 2.4. Biopsy

Surgical debridement of wounds was performed if clinically indicated, as determined by the wound healing attending physician (CEA). If debridement was performed, biopsies, including a small piece of normal skin at the edge of the lesion, were taken. This is standard procedure in our center when evaluating for the presence or absence of vasculitis and is thought to give the highest yield of detecting small vessel vasculitis in vessels away from the base of the ulcer. All specimens were evaluated by an attending pathologist and the primary investigator (VKS), both of whom are experienced at reviewing skin biopsy specimens for the presence of vasculitis. The specimens were graded as to the presence or absence of fibrin plugging, leukocytoclastic vasculitis, or vessel necrosis.

### 2.5. Evaluation for Infection

It is well known that many chronic wounds become colonized with bacteria, but not all colonized wounds are infected. Following standard procedure in the Center for Wound healing, all patients were evaluated at each visit for symptoms or signs of acute infection, based on presence of purulence, odor, ascending erythema, fever, or systemic symptoms. If infection was present, the wound was debrided, and antibiotics were initiated and tailored according to the results of deep cultures taken in the operating room [14]. 

### 2.6. Local Therapy

Patients were treated with aggressive local wound therapy as determined by standardized protocols used by the Center for Wound Healing [14]. Patients with associated macrovascular disease were referred for surgical intervention. Dressings were selected to promote wound healing based on accepted criteria of maintaining high humidity at the wound-dressing interface, removing excess exudate, promoting gaseous exchange, providing thermal insulation, being impermeable to bacteria and other contaminants, and being removable without causing trauma to the wound bed. At each follow-up visit ulcer planimetry was used to assess interval change in ulcer size. Reduction in size at a rate of 10% per week was consistent with healing.

### 2.7. Systemic Therapy

Based on case reports in the literature, open label low-dose, low-molecular-weight heparin (Enoxaparin, 40 mg subcutaneously once daily, Sanofi-Aventis) was used in four patients [9, 10], and darbopoetin alfa (Aranesp, 0.45 mcg/Kg subcutaneously once weekly, Amgen Inc.) was used in one patient [15]. The outcomes of these interventions are reported. 

### 2.8. Pain and Quality of Life Assessment

At the initial visit and upon healing of the wound, patients were asked to complete visual analogue pain score and two quality of life assessments, the Short Form 36 (SF36) which has been validated in many populations including those with scleroderma [16], and diabetic leg ulcers [17] and the Cardiff Wound Impact Schedule (CWIS) which was specifically developed for assessing the impact of leg ulcers on quality-of-life and which has been validated in a population of patients with refractory leg ulcers [18]. Both the SF-36 and the Cardiff Wound Impact Schedule are validated quality-of-life instruments in which the patients' answers to specific questions are scored. The scores are computed using validated formulae to calculate a total score from 0 (worst quality of life) to 100 (best quality of life).

### 2.9. Statistical Analysis

Demographic data was analyzed using descriptive statistics. Interval change in wound percent surface area was calculated at each visit, and wounds were stratified as healed, healing (defined as a reduction of surface area >10% per week), or open (reduction in surface area <10% per week). Quality-of-life and pain scores were analyzed according to wound status (open or healed) at the time the data was recorded, and paired *t*-test was used to analyze these results. Due to the small number of patients being studied and the uncontrolled nature of the interventions in these patients, no statistical analysis was performed on the outcome data. 

## 3. Results

### 3.1. Prevalence of Leg Ulcers in Scleroderma

Between August 2007 and August 2009, 10 of 249 scleroderma patients had active leg ulcers. The prevalence of leg ulcers in our scleroderma population was therefore 4.0%. 

### 3.2. Demographic Features

Of the 10 patients with scleroderma associated leg ulcers, 2 had diffuse scleroderma; 6 had limited scleroderma, and 2 had scleroderma sine scleroderma (Table 1). Consistent with our scleroderma population, 70% were female, and 90% were Caucasian. The age at first ulcer was normally distributed with a mean age of 59.90 years (range, 42 to 76 years, median 59.50 years). 

### 3.3. Disease Duration

The duration of scleroderma at the time of first ulcer development was also normally distributed with a mean age of 18.14 years (range from 4 to 46 years, median 18.5 years). The 2 patients with diffuse scleroderma had shorter disease duration prior to ulcer development (mean 4.05 years ± 0.05) compared to those with limited scleroderma (mean 22.83 years ± 5.612, *P*-value  .0078).

### 3.4. Ulcer Distribution

Ulcers were bilateral in 7 of the 10 patients (70%). Notably, all three of the patients with unilateral lesions had underlying large vessel disease (two with arterial disease and one with venous insufficiency based on Doppler ultrasound measurements). Of the patients with bilateral ulcers, all had lesions in the perimalleolar or anterior ankle region, and two patients additionally had more distal lesions on the feet. 

### 3.5. Biopsy Findings

**Figure 1:**
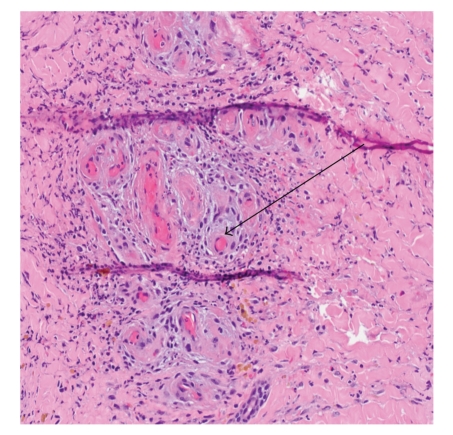
Biopsy of patient 7 showing fibrin occlusive vasculopathy (arrow).

Biopsy specimens were available for review in 5 of the 10 patients (Table 1). All five biopsies showed fibrin plugging and vasculopathy changes as seen in Figure 1. None of the biopsy specimens had evidence of vasculitis.

### 3.6. Antibody Profile

The autoantibody profile is listed in Table 1. Anticentromere antibody was positive in four of the six patients with clinically limited disease. One patient had positive Scl70 antibody, and the other patient was a man with clinically limited scleroderma and positive SSA antibody. Two patients had clinically diffuse scleroderma. One had antitopoisomerase antibody (Scl-70), and another was positive for RNA polymerase III (pol 3). All patients had normal complement levels. 

### 3.7. Antiphospholipid Profile

The results of the antiphospholipid profiles are shown in Table 1. All 10 patients had at least one antiphospholipid profile, and 9 patients had two phospholipid profiles 12 weeks apart, as it is recommended to confirm the presence of antiphospholipid antibodies [19]. Of the 10 patients, 5 had persistently positive antiphospholipid antibodies, giving a prevalence of antiphospholipid antibodies in this group of scleroderma patients with leg ulcers of 50%. Although phospholipid antibody data was not available on the cohort of scleroderma patients without leg ulcers in this study, these rates are higher than reported in the general scleroderma population. Of the 5 patients with persistently positive antibodies, 4 additionally had a history of pregnancy morbidity or vascular thrombosis although they were not on chronic anticoagulation. 

### 3.8. Procoagulant Profile

The genetic procoagulant profile is also listed in Table 1. Homozygous or heterozygous mutation for methyltetrahydrofolate reductase (MTHFR) C677T mutation was seen in 7 of the 10 patients (70%). Plasminogen activator inhibitor gene (PAI-1) mutation was heterozygous positive in 3 patients and homozygous in 1 patient (40%). Factor V leiden and prothrombin gene mutations were not identified in any patient studied. All patients had normal protein C, S, and anti-thrombin III activity. 

### 3.9. Pain and Quality of Life

Pain and quality-of-life data was available in 7 patients enrolled in the CLUE study. 

Pain score was significantly lower in patients with healed wounds (0.6 ± 0.6) compared to those with open lesions (5.025 ± 1.007, *P*  .0351), clearly demonstrating that wound healing correlates with a dramatic improvement in pain. 

The CWIS well-being score was significantly better in the patients with healed wounds (58.84 ± 5.465) compared to those with open wounds (37.93 ± 3.757, *P*.0334). The CWIS physical score was also higher in the patients with healed wounds (86.97 ± 1.57) compared to those with open wounds (59.62 ± 6.295, *P*  .0358). However, there was no difference in social functioning with wound healing. Analysis of the SF-36 data in this small population did not identify significant differences between the healed and unhealed wounds in any of the SF-36 domains. 

### 3.10. Response to Therapy

Due to the association of ulcers with antiphospholipid antibodies and the successful outcomes seen in patients with livedoid vasculopathy, low-dose, low-molecular weight heparin (Enoxaparin, 40 mg subcutaneously once daily, Sanofi-Aventis) was used in 5 patients. Rapid and complete healing was seen in 2 of the patients (patient 1 and patient 6); both of whom had positive antiphospholipid antibodies. Patient 7 just recently commenced therapy with low-dose enoxaparin and to date has demonstrated 50% reduction in ulcer surface area in 3 months. Patient 4 developed bleeding with low dose enoxaparin and had to discontinue the medication. She was subsequently treated with darbopoetin alfa (Aranesp, 0.45 mcg/Kg subcutaneously once weekly, Amgen, Inc.) for anemia, and this resulted in complete healing of the ulcer. Patient 3 had negative antiphospholipid antibodies but homozygous mutation for MTHFR C677T and heterozygous mutation for PAI-1. To date, she has been refractory to low-molecular-weight heparin even at doses of 1 mg/kg twice daily. She remains unhealed after 350 months of follow-up. Pentoxifylline, a xanthine derivative with anti-TNF and fibrinolytic actions, was used in one patient (patient 5) with healing of the ulcers. 

### 3.11. Prognosis of Leg Ulcers in Scleroderma

Of the 10 scleroderma patients with leg ulcers prospectively followed in this study, all had open lesions for ≥3 months. Complete healing has been seen in 6 patients (2 with LMWH, 1 with darbopoetin alfa, 1 with pentoxifylline, 1 with nifedipine, and 1 with arterioplasty), and one patient is responding to LMWH though not completely healed. Ulcers remain refractory to healing in three patients. 

## 4. Discussion

The prevalence of non-healing lower extremity ulcers in our scleroderma population was 4%. In this study all scleroderma patients presenting to the Rheumatology Clinic were evaluated for scleroderma-associated leg ulcers. While there may be a perceived bias because of our special interest in scleroderma-associated lower extremity ulcers, only two patients were identified who were not previously known to have scleroderma both of whom had scleroderma sine scleroderma. Based on this finding, we think that the prevalence reported is likely a true estimate of prevalence of non-healing lower extremity wounds in scleroderma. Furthermore, this highlights the importance of evaluating patients with non-healing wounds for scleroderma even in the absence of overt skin changes. No other studies have specifically evaluated a cohort of scleroderma patients for non-digital ulcer prevalence, but our data are in line with that reported by Alivernini et al. [2]. The cumulative incidence of diabetic leg ulcers over a 5-year period has been reported at 5.8% [3], suggesting that the frequency of leg ulcers in scleroderma approaches that seen in diabetes. 

Biopsy studies were available in 50% of patients in this study. We did not identify vasculitis in any of the biopsied wounds. While any biopsy always carries a risk of sampling error, we believe that biopsies which include the subcutaneous tissue and a perimeter of normal skin at the edge of the wound are usually sufficient to confirm the presence or absence of vasculitis [14]. 

Our study design had significant limitations since we do not have funding or IRB approval to screen our entire scleroderma population for prevalence of antiphospholipid antibodies and genetic prothrombotic states. This limits our ability to draw firm conclusions regarding the associations with lower extremity ulcers. 

Although our study was limited due to the sample size and study design, we were able to demonstrate that scleroderma associated ulcers are refractory to usual wound care therapies. Additionally, while the quality of life questionnaires administered in this study work best in large population studies, our data do suggest that presence of open wounds in scleroderma adversely impact quality-of-life over and above the underlying scleroderma. 

The prevalence of antiphospholipid antibodies in our cohort of patients with ulcers was higher than that reported in the general scleroderma population (50% compared to between 3.3 and 12%) [20–22]. Several other studies suggest an association between antiphospholipid antibodies and lower extremity ulcers in scleroderma. Lupus anticoagulant has been identified as a strong predictor of ulcer presence in scleroderma patients (OR 7.2) [2]. Furthermore, cutaneous ulcers are more frequent in scleroderma patients with antiphospholipid antibodies than those without (63% compared to 39%) [22]. Finally, a study reporting a series of eight patients with concomitant scleroderma and antiphospholipid syndrome identified three patients (37.5%) with associated leg ulcers [23], a much higher prevalence than we found in our more general scleroderma population. 

MTHFR C677T heterozygous or homozygous mutation was also higher than expected in our cohort of scleroderma patients with leg ulcers. We found a prevalence of 60% for the heterozygous mutation and 10% for the homozygous mutation whereas in the general scleroderma population 49% expressed wild type (no mutation), 36% were heterozygous, and 15% were homozygous for the mutation [24]. Our population of patients with scleroderma associated leg ulcers had a prevalence of the plasminogen activator inhibitor gene (PAI-1) mutation of 40%. This is on a par with frequencies of this gene mutation in other populations, with the 4G allele being reported at a frequency of 62% in healthy pregnant women [25]. 

The outcome of the small number of patients treated with low-molecular-weight heparin therapy is promising. When tolerated, we found a 50% complete healing rate. Furthermore, response did not require full therapeutic doses of heparin, suggesting that, heparin may be acting via antiphospholipid-dependent pathways, such as complement activation and fibrinolysis, rather than purely through its anticoagulant effect as has been reported for antiphospholipid-associated pregnancy losses [12]. Only one patient in our study was treated with darbopoetin alfa but this resulted in complete healing of her ulcer. A similar response has been reported in one other case report [15]. The erythropoietin analogues are increasingly recognized as stimulators of angiogenesis pathways, and therefore these pathways may merit further investigation in scleroderma [26]. 

## 5. Conclusions

Lower extremity ulcers are seen in 4% of scleroderma patients and cause pain and morbidity over and above that of the scleroderma. In this small study we identified higher than expected frequency of antiphospholipid antibodies and MTHFR mutation. We recommend that scleroderma patients who develop leg ulcers should undergo prothrombotic evaluation. Clearly this small uncontrolled study is insufficient to draw clear conclusions as to etiology of delayed wound healing in scleroderma. However, lower extremity ulcers represent a challenging clinical problem in scleroderma and further studies into their pathogenesis and potential therapies may yield new insights into the vasculopathy of scleroderma at a cellular and molecular level. 

## Figures and Tables

**Table 1:** Features and outcomes of patients with scleroderma associated leg ulcers.

Pt			1	2	3	4	5	6	7	8	9	10
Sex			M	M	F	F	M	F	F	F	F	F

Race			C	C	C	C	C	H	C	C	C	C

SSc clinical subtype			Sine	Limited	Limited	Diffuse	Limited	Limited	Diffuse	Limited	Limited	Sine

Disease duration at time of ulcer development (years)			NA	20	17	4.1	4	46	4	26	24	NA

Scleroderma-specific antibody			U3RNP	Centromere	Centromere	RNA Pol3		Centromere	Scl70	Centromere	Scl70	Centromere

Other Antibodies			Nucleolar ANA; RF	Speckled ANA		Speckled ANA	SSA	Nucleolar ANA	Speckled ANA	Speckled ANA		

Other scleroderma features			GI dysmotility, GERD	GI dysmotility, GERD, SICCA, limited skin	GI dysmotility, SICCA, limited skin, joint contractures.	Diffuse skin, SICCA, arthritis, interstitial lung disease, GI dysmotility	SICCA, limited skin, joint contractures	GERD, Telangiectasias	Diffuse skin, interstitial lung disease, GI dysmotility, SICCA, calcinosis	Limited skin, joint contractures, SICCA, GERD, GI dysmotility, calcinosis	Limited skin, arthritis, pulmonary hypertension, GERD, SICCA, GI dysmotility	Calcinosis

Ulcer location			Left leg	Left medial malleolus	Bilateral malleoli and right posterior ankle	Bilateral malleoli	Bilateral Toes, dorsal foot and heel	Bilateral lateral calf	Right medial malleolus, left dorsal foot	Bilateral malleoli	Left lateral malleolus	Bilateral toes and bottom of feet

Venous insufficiency on doppler US			−	−	−	−	−	−	−	−	+	−

Arterial Disease on ABPI			+	+	−	−	−	−	−	−	−	−

												
SCREEN 1	LAC		+	−	−	_+_	−	−	+	−	−	−
	*β*-2GPI (normal <10 U/mL)	IgG	39	<10	<10	<10	<10	<10	41	<10	<10	<10
		IgA	<10	13	<10	<10	<10	100	23	<10	<10	<10
		IgM	<10	<10	<10	<10	<10	<10	<10	<10	<10	<10
	ACL (normal <10 U/mL)	IgG	<10	<10	<10	45	<10	<10	23	<10	<10	<10
			IgA	<10	<10	<10	<10	<10	<10	<10	<10	<10	<10
			IgM	<10	<10	<10	<10	<10	<10	<10	<10	<10	<10

												
SCREEN 2	LAC		+	−	−	+	−	−	+	−	−	NT
	*β*-2GPI (normal <10 U/mL)	IgG	23	<10	<10	19	<10	<10	<10	<10	<10	NT
		IgA	<10	20	<10	<10	<10	100	<10	<10	<10	NT
		IgM	<10	<10	<10	<10	<10	<10	<10	<10	<10	NT
	ACL (normal <10 U/mL)	IgG	<10	<10	<10	20	<10	<10	<10	<10	<10	NT
		IgA	<10	<10	<10	14	<10	<10	<10	<10	<10	NT
		IgM	<10	<10	<10	<10	<10	<10	<10	<10	<10	NT

												
Summary APLprofile			+	+	−	+	−	+	+	−	−	−

												
Genetic procoagulant profile	MTHFR		0	1	2	1	1	1	0	1	1	NT
	PAI-1		0	1	1	0	2	0	1	0	0	NT
	Prothrombin Gene		0	0	0	0	0	0	0	0	0	NT
	FVL		0	0	0	0	0	0	0	0	0	NT

Summary Genetic procoagulant profile			−	+	+	+	−	+	+	+	+	NT

Biopsy			Fibrin occlusive vasculopathy	No biopsy	Fibrin occlusive vasculopathy	Fibrin occlusive vasculopathy	No biopsy	No biopsy	Fibrin occlusive vasculopathy	Fibrin occlusive vasculopathy	No biopsy	No biopsy

Treatment			Enoxaparin 40 mg daily, arterioplasty	Arterioplasty	Enoxaparin 1 mg/Kg twice daily	Enoxaparin stopped due to bleeding Darbepoetin alfa	Pentoxifylline 400 mg three times per day	Enoxaparin 40 mg daily	Enoxaparin 40 mg daily	None	Venous surgery pending	Healed with Nifedipine

Outcome			Healed	Healed	Not healed	Healed	Healed	Healed	50% healing in 3 months	Not healed	Not healed	Healed

Total duration of ulcer (months)			36	6	350	10	6	23	36	3	5	6

Time to healing after initiation of therapy			4	6	—	3	6	4	—	—	—	3

GERD: Gastroesophageal reflux disease; SICCA: dryness of the conjunctiva and cornea and dryness of the mouth; GI dysmotility: gastrointestinal dysmotility; ACL: anticardiolipin antibodies; *β*-2GP1 Ab: Beta-2 Glycoprotein I antibodies; LAC: lupus anticoagulant; MTHFR: Methyltetrahydrofolate reductase mutation; PAI-1: Plasminogen Activator Inhibitor-I mutation; FVL: Factor V Leiden mutation;

For gene mutation results 1: heterozygous mutation, 2: homozygous mutation; NT: not tested.
